# Blockade of TIM3 relieves immunosuppression through reducing regulatory T cells in head and neck cancer

**DOI:** 10.1186/s13046-018-0713-7

**Published:** 2018-03-05

**Authors:** Jian-Feng Liu, Lei Wu, Lei-Lei Yang, Wei-Wei Deng, Liang Mao, Hao Wu, Wen-Feng Zhang, Zhi-Jun Sun

**Affiliations:** 1 The State Key Laboratory Breeding Base of Basic Science of Stomatology (Hubei-MOST) & Key Laboratory of Oral Biomedicine Ministry of Education, School & Hospital of Stomatology, Wuhan University, Wuhan, China; 2 Department of Oral Maxillofacial-Head Neck Oncology, School and Hospital of Stomatology, Wuhan University, Wuhan, China

**Keywords:** Head and neck cancer, Immunosuppression, Immunotherapy, Immune checkpoint, Tregs, Macrophages

## Abstract

**Background:**

T-cell immunoglobulin mucin 3 (TIM3) is a negative immune checkpoint and plays a crucial part in tumor-induced immune suppression. However, the mechanism of TIM3 in regulating immunosuppression in head and neck squamous cell carcinoma (HNSCC) was still not quite clear.

**Methods:**

We carried out the immunohistochemistry staining of HNSCC tissue microarrays. Through quantification of the histoscore, we performed the correlation analysis among the TIM3, Galectin-9, Foxp3, CD68 and CD163. The effects of TIM3 on regulatory T cells (Tregs) and macrophages were detected by utilizing the *Tgfbr1/Pten* 2cKO HNSCC mouse model. Flow cytometry were used to analysis the percent of Tregs, macrophages and IFN-γ.

**Results:**

We demonstrated the close association among TIM3/Galectin-9 pathway, regulatory T cell marker (Foxp3) and macrophage marker (CD68, CD163) in human HNSCC. In the transgenic HNSCC mouse model, blockade of TIM3 by the anti-TIM3 monoclonal antibody induced a reduction of CD4^+^CD25^+^Foxp3^+^ Tregs. Meanwhile, the population of TIM3^+^ Tregs was also decreased. However, the population of CD206^+^ macrophages was not significantly declined. The increased IFN-γ production on CD8^+^ T cells in anti-TIM3 treatment mice showed that the antitumor immune response was enhanced through suppression of these negative immune factors.

**Conclusions:**

The present study demonstrated that TIM3 was associated with the immunosuppression in HNSCC. And targeting TIM3 can enhance anti-tumor immune response by decreasing Tregs in HNSCC.

## Background

Head and neck squamous cell carcinoma (HNSCC) is one of the most common malignancies around the world [1]. It mainly takes place in the oral cavity, mouth pharynx, larynx and laryngopharynx, and is characterized by local invasion and metastasis [2]. Tobacco and alcohol consumption are considered as the major cause of HNSCC, and HPV infection has emerged as another important risk factor in recent years [3]. Although treatments have improved and the use of targeted medicines (such as cetuximab) combined with radiotherapy have improved the living quality and prognosis of patients, the 5 year overall survival rate is still approximately 50% [4].

Recent studies have indicated that the development of HNSCC is closely related to immunosuppression and immune escape. The aberrant activities of T lymphocytes, B lymphocytes, dendritic cells (DC), macrophages, NK cells and various cytokines are involved in the initiation, promotion and progress of HNSCC [5]. Regulatory T cell (Treg) is a subset of CD4^+^ T cells and serves as an inhibitor of the antitumor immune response [6]. Tregs can inhibit immune effector cells by releasing suppressive cytokines [7]. The transcription factor Foxp3 is a necessary marker for activity of Treg cells [8]. Recent evidence has demonstrated that Treg activity is increased in HNSCC patients, but the prognostic value of Treg in HNSCC is still controversial [9].

In addition to Tregs, macrophages also take a part in tumor initiation and promotion. Macrophages have been sorted into two main subgroups: classically activated macrophages (M1) and alternatively activated macrophages (M2) [10]. M1 macrophages have anti-tumor effects on tumorigenesis, while M2 macrophages promote tumor development by inhibiting tumor-specific immune response [11]. M2 macrophages in solid tumors are comparatively more than M1 macrophages [12]. CD206 is one of the surface molecules specific to M2 macrophages. In addition, Zhu et al. have suggested that CD206 expression was related to the prognosis of hepatocellular carcinomas [13].

T-cell immunoglobulin mucin 3 (TIM3), an important immune checkpoint protein, was initially shown to be expressed on CD4^+^ Th1 cells and CD8^+^ T cells [14]. Then, studies successively demonstrated TIM3 expression on macrophages, monocytes and CD11b^+^ DCs [14–16]. Although several molecules have been reported bind to TIM3 [16–18], Galectin-9 is considered as the major ligand [19]. By binding to Galectin-9, TIM3 induces T lymphocytes exhaustion or apoptosis [20]. While blockade of TIM3 could promote IFN-γ-mediated antitumor immunity of T cells [21]. A research showed that TIM3 was expressed on Tregs and correlated with rheumatoid arthritis activity [22]. An in vitro experiment suggested that TIM3 on Tregs was correlated with tumor size of ovarian carcinoma [23]. Moreover, TIM3 could be upregulated by stimuli and may be associated with macrophage activity [24]. Our previous study has demonstrated TIM3 is overexpressed in HNSCC and is associated with myeloid-derived suppressor cells MDSCs [25]. However, the role of TIM3 in modulating Tregs and macrophages in HNSCC is still unknown.

We have previously demonstrated the function of TIM3 in regulating effector T cells in HNSCC [25]. In this study, we explored the role of TIM3 in regulating Tregs and macrophages in HNSCC. HNSCC tissue arrays were used to analyze the association among the TIM3/Galectin-9 signal, the Treg marker (Foxp3) and macrophage markers (CD68, CD163). By utilizing an HNSCC mouse model, we explored TIM3 function in regulating Tregs and M2 macrophages.

## Methods

### Patient samples and HNSCC tissue microarray

The Medical Ethics Committee of the School and Hospital of Stomatology Wuhan University approved this study. Human HNSCC tissue samples were acquired from the Hospital of Stomatology Wuhan University. All the patients accepted the informed consent before the surgery. The HNSCC samples, including 27 normal mucosa, 122 primary HNSCC, were used to construct tissue microarrays and applied to immunohistochemistry staining.

### Immunohistochemistry

The immunohistochemistry staining of sections were performed according to the procedure as previously described [25]. The following antibodies were used: TIM3, Galectin9, Foxp3 (Cell Signaling Technology, USA), CD68 (Zymed, China), or CD163 (CW Biotech, China).

### Animals

The animal experiments were performed under the guidelines of the Institutional Animal Care and Use Committee of Wuhan University. The spontaneous HNSCC mouse model is a transgenic mouse with a combined *Tgfbr1/Pten* knockout (*K14-Cre*^ERtam+/−^;*Tgfbr1*^flox/flox^; *Pten*^flox/flox^) and with the background of CD1/129/FVBN/ C57/BL/6. Transforming Growth Factor-β (TGF-β) and components of the PTEN/PI3K/Akt signal pathways are the most common mutation molecules associated with HNSCC progress. *Tgfbr1* and *Pten* knockout by tamoxifen induction in head and neck epithelium of mice could result in the occurrence of squamous cell carcinoma with full penetrance. This *Tgfbr1/Pten* 2cKO mouse model is immunocompetent, and it is suitable for cancer immunotherapy research. After five sequential days of tamoxifen treatments by oral gavage, *Tgfbr1/Pten* were knocked out in epithelium of oral cavity and head-neck region. The course of tamoxifen usage was illustrated as before [26]. During the induction process, squamous cell carcinoma occurred in head-neck region of the mice. This mouse model was maintained and genotyped as the previous description [26].

### Mice treatment

After tamoxifen induction for 5 days, the mice were divided into control group (*n* = 6) and anti-TIM3 group (*n* = 6) at random. Rat isotype IgG2a was applied to the control group. The prophylactic administration of isotype IgG2a (clone 2A3) or anti-TIM3 (RMT3–23) (BioXCell, West Lebanon, NH) in mice was carried out by intraperitoneal injections (100 μg i.p.) for 3 days since day 12 and then once a week for the following weeks. The tumor size of mice was measured every five days. Finally, the mice were executed by euthanasia.

### Flow cytometry

The single cell suspensions were obtained from the draining lymph node and the spleens and then stained with antibodies. The following antibodies were used: PE/Cy7-conjugated CD8, FITC-conjugated CD11b, CD4, PE-conjugated CD25, F4/80 and Foxp3 (eBioscience, San Diego, CA). APC-conjugated TIM3 and TIGIT, PE-conjugated CD206, BV-421 conjugated PD1 and LAG3, and PE/Cy7-conjugated CTLA4 (BioLegend, San Diego, CA). 7AAD (Invitrogen) was applied to exclude dead cells. The CytExpert software (Beckman Coulter, CA, USA) was used for flow cytometry analysis.

### Scoring system

Aperio Scan Scope CS scanner (Vista, CA, USA) was used to scan the HNSCC tissue microarray. The interested area of the section was chosen for quantification. The score of the IHC staining was quantified with background subtraction using Aperio Quantification software (Version 9.1). The histoscore of the nucleus and membrane staining were worked out using the following formula for the percentage of cells with different positive degrees: (3+) × 3 + (2+) × 2 + (1+) × 1 [27]. Histoscores were translated to numerical scores from − 3 to 3 using Microsoft Excel. Cluster 3.0 was used to perform the hierarchical analysis. Finally, the cluster picture was completed with Java TreeView 1.0.5.

### Statistical analysis

Statistical data analysis was performed by utilizing the GraphPad Prism 6 software (La Jolla, CA) and was shown as the mean values ± SEM. The Mann-Whitney test was used to analyze the differences between two different groups. And the value of *Cohen’s d* of each data have been calculated to confirm the significant difference between two different groups. The two-tailed Pearson correlation was used to evaluate the expression relevance of TIM3, Galectin-9, Foxp3, CD68 and CD163. Statistical significance was defined as *P* value < 0.05.

## Results

### TIM3/Galectin-9 pathway was correlated with the expression of Foxp3, CD68 and CD163 in HNSCC

**Fig. 1:**
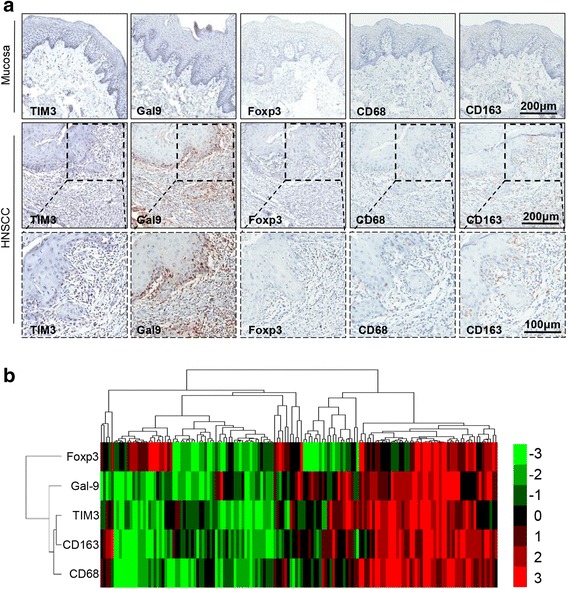
TIM3, Galectin-9 and Foxp3 expression in HNSCC. **a** The representative IHC photographs of TIM3, Galectin-9 and Foxp3 expression in human normal oral mucosa and HNSCC tissue. **b** Hierarchical clustering presents the correlation among TIM3, Galectin-9 and Foxp3 in the human HNSCC tissue array

**Fig. 2:**
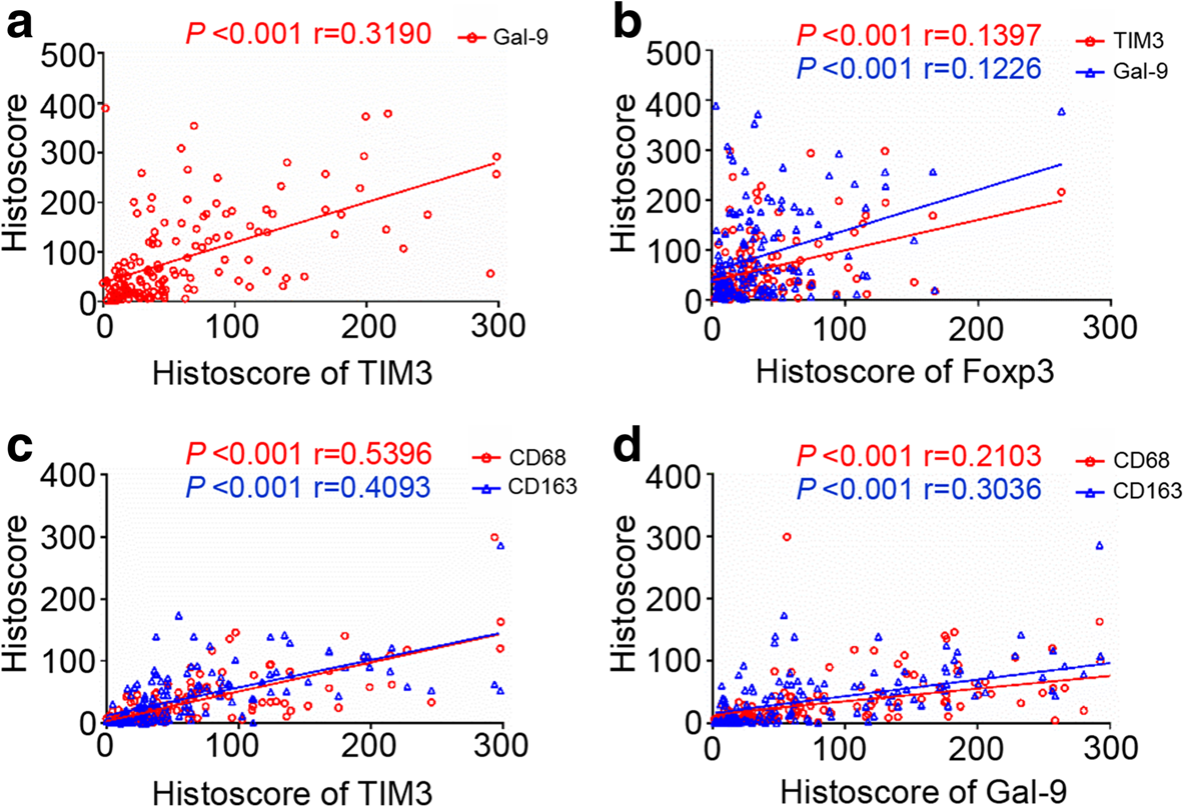
Correlation analysis of TIM3, Galectin-9, Foxp3, CD68 and CD163 protein expression in the human HNSCC tissue array. **a** Correlation of TIM3 with Galectin-9. **b** Correlation of Foxp3 with TIM3 and Galectin. **c** Correlation of TIM3 with CD68 and CD163. **d** Correlation of Galectin-9 with CD68 and CD163

To evaluate the TIM3/Galectin-9 signaling pathway in HNSCC, we detected TIM3 and Galectin-9 expression in human HNSCC tissue arrays. The IHC photographs demonstrated that TIM3 was specifically expressed on immune cells in the tumor stroma. As the ligand of TIM3, Galectin-9 was not only expressed on tumor cells of invasive front but also on immune cells in the tumor stroma. We also examined the Treg marker (Foxp3) and macrophage markers (CD68 and CD163) in the HNSCC tissue array. These markers (Foxp3, CD68, and CD163) were expressed on immune cells in the tumor stroma (Fig. 1a). The cluster analysis showed the IHC scores of TIM3, Galectin-9, Foxp3, CD68 and CD163 for each patient and the correlation of their expression in HNSCC patients (Fig. 1b). Further correlation analysis shows that TIM3 expression was closely associated with Galectin-9 expression (Fig. 2a). Foxp3 expression was correlated with TIM3 and Galectin-9 expression (Fig. 2b). Additionally, the expression of TIM3 and Galectin-9 were significantly correlated with CD68 and CD163 expression (Fig. 2c, d).

### Percent of Tregs and CD206^+^ macrophages increased in the HNSCC mouse model

**Fig. 3:**
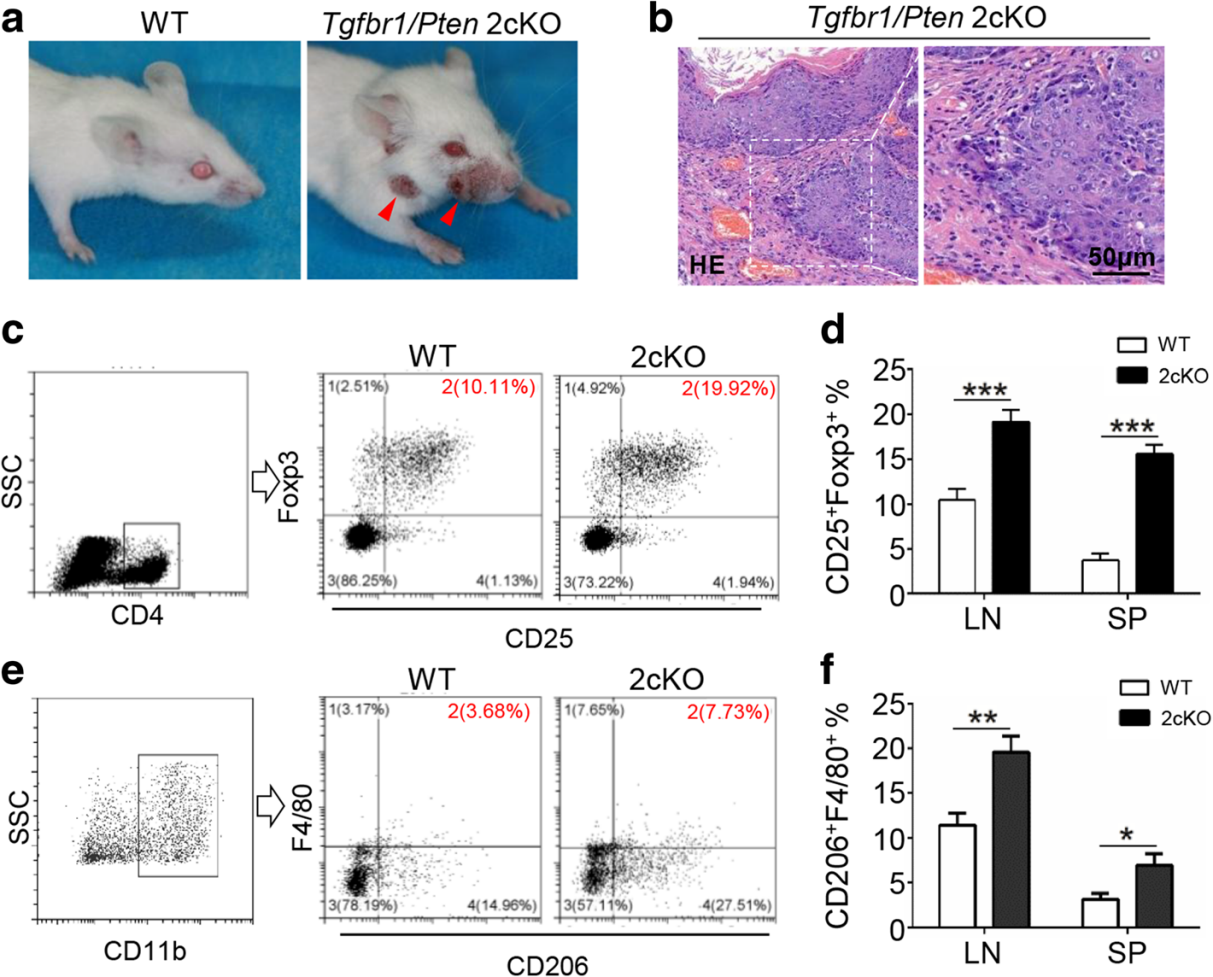
The number of TIM3^+^ Tregs increased in the *Tgfbr1/Pten* 2cKO HNSCC mouse model. **a** Photographs of a wild-type mouse and a *Tgfbr1/Pten* 2cKO HNSCC mouse. **b** HE staining of squamous cell specific tumor tissue in the 2cKO HNSCC mouse model. **c** Flow cytometry analysis photographs of CD25^+^Foxp3^+^ cells gated from CD4^+^ T cells. **d** The percent of Tregs from the draining lymph node (LN) and spleen (SP) in WT (*n* = 6) and HNSCC (*n* = 6) mice. **e** Flow cytometry analysis photographs of CD206^+^F4/80 cells in the CD11b^+^ cell populations. **f** The ratio of CD11b^+^CD206^+^F4/80 macrophages from LN and spleen SP in WT and HNSCC mice. (mean ± SEM, *P < 0.05, **P < 0.01, ***P < 0.001, Mann-Whitney test)

The phenotypic and pathological features of head and neck squamous cell carcinoma of *Tgfbr1/Pten* 2cKO mouse model are shown in Fig. 3a and b. Tregs and M2 macrophages are considered as the immunosuppression-mediated cells which suppress the immune response to cancer cells. We detected the percent of CD4^+^CD25^+^Foxp3^+^ Tregs and CD11b^+^F4/80^+^CD206^+^ macrophages (M2) in the HNSCC mouse model. The flow cytometry analysis showed that in the HNSCC mice, the percent of Tregs was increased compared with that of wild-type (WT) mice (Fig. 3c and d). Meanwhile, the percent of CD11b^+^F4/80^+^CD206^+^ macrophages was also increased in the HNSCC mouse model (Fig. 3e and f). These results showed that the negative immune regulators, Tregs and M2 macrophages, accumulate in the development of HNSCC.

### Blockade of TIM3 induced a decrease of Tregs in HNSCC mice

**Fig. 4:**
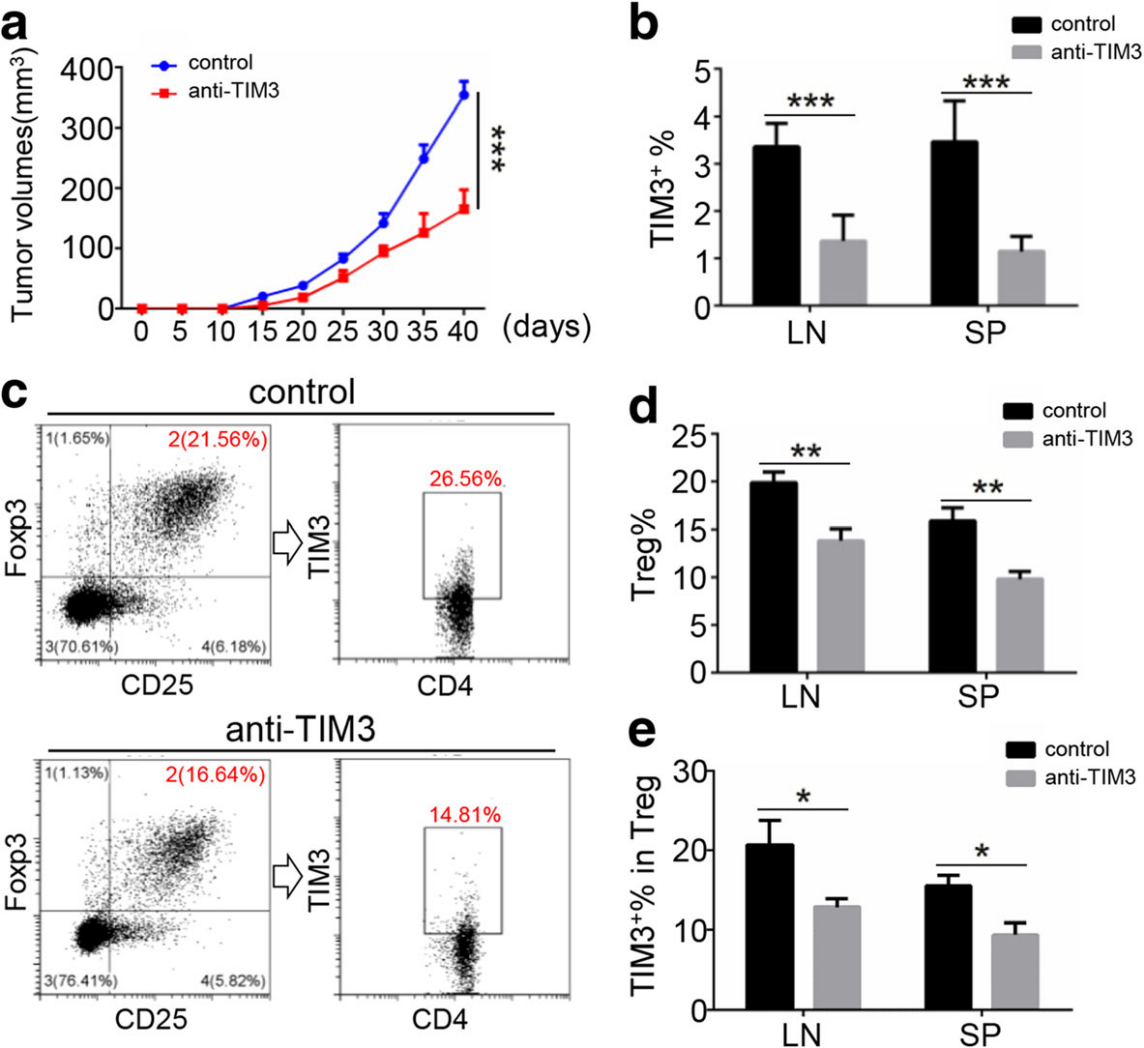
Blockade of TIM3 reduced the number of TIM3^+^ Tregs. **a** The tumor growth curve of each group. **b** Percent of TIM3^+^ cells from the draining lymph node (LN) and spleen (SP) in the anti-TIM3 therapy group (*n* = 6) and control group (*n* = 6). **c** CD4^+^ T cells were gated from CD25^+^ Foxp3^+^; then, TIM3^+^ cells in CD4^+^CD25^+^Foxp3^+^ Treg population was assessed. **d** Percent of CD25^+^Foxp3^+^ Tregs in the CD4^+^ T cell population from LN and SP of anti-TIM3 therapy group (*n* = 6) and control group (*n* = 6). **e** Percent of TIM3^+^ cells in the CD4^+^CD25^+^Foxp3^+^ Treg population (mean ± SEM, *P < 0.05, **P < 0.01, ***P < 0.001, Mann-Whitney test)

We next explored the role of TIM3 in immune suppression by using the HNSCC mouse model. The tumors with the anti-TIM3 therapy grew at a slower rate than those of the control group (Fig. 4a). Through flow cytometry analysis, we found that TIM3 expression was reduced in the anti-TIM3 group (Fig. 4b). Furthermore, we examined the population of Tregs in each group. The results showed that the percent of CD25^+^Foxp3^+^ cells in CD4^+^ T cells was significantly decreased in anti-TIM3 therapy mice compared with that of control mice (Fig. 4c and d), which means that Tregs were reduced in response to TIM3 blockade. Furthermore, these CD4^+^CD25^+^Foxp3^+^ Tregs were labeled with TIM3. Interestingly, the percent of TIM3^+^ Tregs was also down-regulated in the anti-TIM3 therapy group (Fig. 4e). It suggested that TIM3 may participate in the differentiation of Tregs, and that the blockade of TIM3 induced a decline in the number of Tregs.

### Impact of TIM3 blockade on immune checkpoints and macrophages

**Fig. 5:**
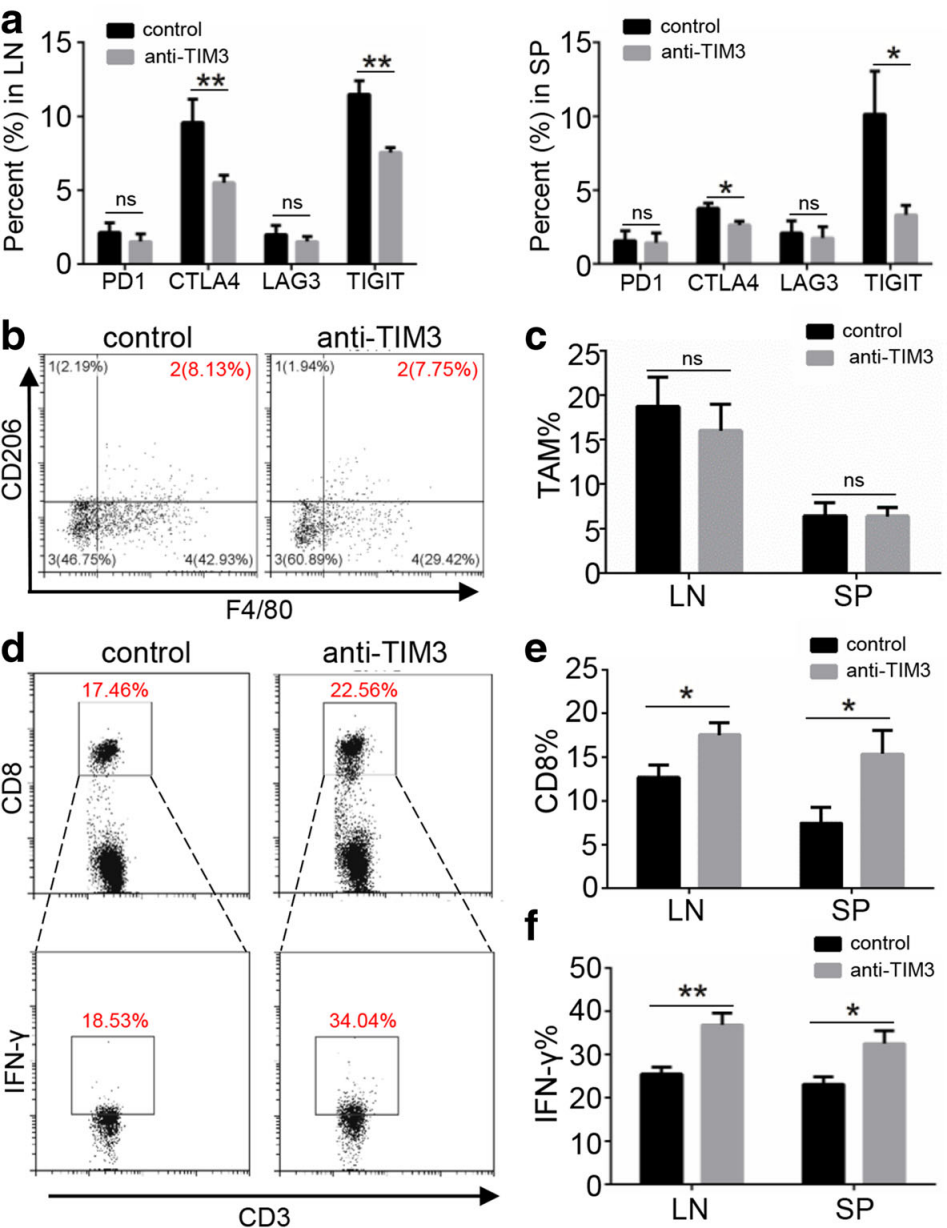
Impact of TIM3 blockade on immune checkpoints, M2 macrophages and IFN-γ production. **a** Percent of PD1, CTLA4, LAG3 and TIGIT expressed in the LN and SP of each group. **b** F4/80^+^CD206^+^ macrophages gated from the CD11^+^ cells in each group. **c** Percent of CD11b^+^F4/80^+^CD206^+^ macrophages in each group. **d** The flow cytometry photos of CD8^+^ T cells and IFN-γ^+^ production on CD8^+^ T cells (gated from CD3^+^ cells) in LN and SP of each group. **e** Percent of CD8^+^ T cells in each group. **f** Percent of IFN-γ^+^ cells in CD8^+^ T cells in each group (mean ± SEM, *P < 0.05, **P < 0.01, ***P < 0.001, Mann-Whitney test)

Immune checkpoints play a vital role in T cell dysfunction and exhaustion. Here, the expression levels of PD1, CTLA4, LAG3 and TIGIT were detected by flow cytometry. The expression levels of PD1 and LAG3 were not changed significantly, while the expression levels of CTLA4 and TIGIT were significantly decreased (Fig. 5a). Moreover, since CD206 is considered an important marker of M2 macrophages, we examined CD11b^+^F4/80^+^CD206^+^ macrophages. However, flow cytometry analysis demonstrated that the number of CD11b^+^F4/80^+^CD206^+^ macrophages was not substantially decreased by blocking TIM3 (Fig. 5b and c).

### Blockade of TIM3 promoted IFN-γ production on CD8 T cells

To verify whether the inhibition of Tregs and immune checkpoints by blocking TIM3 enhanced the anti-tumor immune response, we assessed the IFN-γ expression on CD8^+^ T cells. Flow cytometry analysis showed that the percent of CD8^+^ T cells was significantly increased in the anti-TIM3 therapy mice compared with that in the control mice (Fig. 5d, e). Furthermore, the IFN-γ production on CD8^+^ T cells was also remarkably elevated by anti-TIM3 therapy (Fig. 5f). TIM3 blockade enhanced the antitumor immune response in HNSCC mouse model.

## Discussion

The immune system acts as a supervisor during the initiation and development of HNSCC. Suppression of the immune system leads to the tumor escape [5]. Tumor cell co-option of immune checkpoints act as the major pathways of immune suppression and immune evasion in cancer [28]. Recent evidence has demonstrated that TIM3/Gal9 is an important inhibitory pathway in the immune response of cancer [29]. TIM3 has been proved to be expressed on multiple immune cells, and blocking TIM3/Gal9 has an effect on various immune cells, such as effector T cells, Tregs, macrophages and monocytes [20, 22, 30, 31]. Our previous study has identified the overexpression of TIM3 in HNSCC patients and the association of TIM3 expression with MDSCs [25]. Evidences showed a general increase in the number of circulating and infiltrating Tregs in HNSCC patients [32, 33]. However, the actual association among TIM3, Tregs and macrophages is not very clear in HNSCC. In this study, we determined that the TIM3/Galectin-9 pathway is closely related to the expression of the Treg marker (Foxp3) and macrophage markers (CD68 and CD163) in a HNSCC tissue array.

The canonical Tregs is a subset of T lymphocyte identified by CD4^+^CD25^+^Foxp3^+^. Tregs can restrain CD8^+^ T cell activation and inflammation through direct contact or production of TGF-β and interleukin (IL)-10 [34]. Early research on ovarian carcinoma demonstrated that blockade of TIM3 reverted Treg-mediated immune suppression [35]. We found that in the transgenic HNSCC mice, the number of Tregs was elevated compared with that of WT mice, while blockade of TIM3 induced a decrease in the Tregs population. Thus, the Treg-mediated inhibition of the immune response mediated was neutralized. Interestingly, the number of TIM3^+^ Tregs was also reduced by anti-TIM3 therapy, suggesting that TIM3 may participate in the regulation of Tregs. Coincidentally, recent studies have shown that TIM3 participated in the regulation of Tregs. Sun et al. observed that the number of TIM3^+^ Tregs was correlated with rheumatoid arthritis activity. Moreover, IL-10 expression on TIM3^+^ Tregs was higher than TIM^−^ Tregs [22]. In hepatitis C viral infections, TIM3 was found to be expressed on Tregs and to regulate the balance between Tregs and effector T cells. And it has been reported that TIM3^+^ Tregs represent the highly suppressive Tregs due to their high production of IL-10, perforin, granzyme A and granzyme G [36]. These findings suggest that TIM3 acts as a vital regulator of Tregs and affects the function of Tregs.

TIM3 has been shown to be involved in regulating the macrophage activity [30]. Zhang et al. reported that TIM3 expression is increased on macrophages in autoimmune diseases. In addition, increased TIM3 expression on M2 macrophages participated in immune regulation by inhibiting macrophage activation [37]. Another research also indicated that the upregulation of TIM3 expression on M2 macrophages in mice mediated the anti-inflammatory response [38]. However, in the present study, blocking TIM3 did not reduce the number of CD11b^+^F4/80^+^CD206^+^ (M2) macrophages significantly in the HNSCC mouse model. This may be due to the limited expression of TIM3 on CD11b^+^F4/80^+^CD206^+^ macrophages in this mouse. The negative immune checkpoints (such as PD-1, LAG3, CTLA4) also play vital roles in immune suppression through multiple pathways in HNSCC development. In the *vivo* study, although PD-1 and LAG3 expression levels were not obviously decreased by TIM3 blockade, CTLA4 and TIGIT expression levels were significantly decreased. CTLA4 acts as a negative regulator of T cell activation and the maintenance of T cell homeostasis [39]. TIGIT is expressed on memory T cells, Tregs and NK cells, and can suppress the activation of T cells [40]. Thus, the downregulation of CTLA4 and TIGIT may alleviate the inhibition of T cell activation and strengthen the immune response. In addition to these mechanisms, the reduced expression of TIM3 on T cells can directly increase IFN-γ production and enhance the anti-tumor immune effect.

## Conclusion

Taken together, HNSCC is a malignant tumor characterized by a substantially suppressed immune system. There are various mechanisms that contribute to the failed anti-tumor immune response. We showed that TIM3 participates in the regulation of Tregs, and that blockade of TIM3 relieves the immune suppression by reducing Treg activation and decreasing CTLA4 and TIGIT in HNSCC, supporting the therapeutic value of anti-TIM3 treatment in HNSCC.

## Abbreviations

DC: Dendritic cells
HNSCC: Head and neck squamous cell carcinoma
IL-10: Interleukin-10
MDSCs: Myeloid-derived suppressor cells
TGF-β: Transforming Growth Factor-β
TIM3: T-cell immunoglobulin mucin 3;
Tregs: Regulatory T cells
WT: Wild type
